# Bat Assemblage in an Oil Palm Plantation from the Colombian Llanos Foothills

**DOI:** 10.21315/tlsr2021.32.1.3

**Published:** 2021-03-31

**Authors:** María Alejandra Cely-Gómez, Dennis Castillo-Figueroa, Jairo Pérez-Torres

**Affiliations:** Laboratorio de Ecología Funcional, Unidad de Ecología y Sistemática, Departamento de Biología, Pontificia Universidad Javeriana, Bogotá, Colombia

**Keywords:** Chiroptera, Orinoquía, Oil Palm, Bats, Assemblages

## Abstract

The surge of oil palm production in the Neotropics has become a major concern about the potential impacts on biodiversity. In the Colombian Orinoquia, which has shown a massive landscape transformation due to the growth of oil palm plantations, the effects of oil palm agriculture on bats in this region have not been studied up to date. To understand the impact of habitat conversion on bat diversity, we characterised bat assemblages in secondary forest and palm plantations in the Colombian Llanos foothills (Meta, Colombia). We captured 393 individuals (forest = 81, plantation = 312) of 18 species and 3 families. The forest cover presented three exclusive species while the plantation had five. Species diversity (*q*^1^) and evenness (*J*′) were higher in the forest compared to the plantation. These differences derived from the increase in abundances of generalist species (*Artibeus* sp., *Carollia* spp.) in the plantation. Despite the habitat simplification caused by oil palm plantations, this monoculture provides a cover that is used by some bats, decreasing their risk of predation and allowing movement between patches of forest habitat as steppingstones. Maintaining forest cover in agricultural landscapes favours diversity by generating a “spillover effect” of the forest towards plantations, which in the case of some bats contributes to the reduction of species isolation and the maintenance of ecosystem services provided by them. It is important to improve management practices of oil palm plantations to minimise negative impacts on biodiversity, considering the expansion of this productive system and the scarcity of protected areas in this region.

**Highlights**

- There were no differences in bat richness (*q*^0^) between secondary forest and oil palm plantations, but the former showed higher evenness (*J′*) and diversity (*q*^1^).
- Generalist species (*Artibeus* sp., *Carollia* spp.) increased their relative abundance in oil palm plantations, as have been shown in other agricultural systems.
- Exclusive species were found for secondary forest and oil palm plantations and abundance of shared species varied in each coverage.

## INTRODUCTION

Neotropical bats present high variability in species richness, abundance, diet and morphology, which can influence ecological interactions such as pollination, seed dispersal and regulation of insect populations central to multiple ecosystem services (Park 2014; Kasso & Balakrishnan 2013; Kunz *et al*. 2011; Cleveland *et al*. 2006). Due to this, bats have been studied in different Neotropical ecosystems under diverse anthropogenic contexts, where the assemblages have revealed changes in species richness or abundance according to elevation (Castaño *et al.* 2018; Bejarano-Bonilla *et al.* 2007; McCain 2007), geographical region (Morales-Martínez *et al.* 2018) and land use management (Montaño-Centellas *et al.* 2015; Ortegón-Martínez & Pérez-Torres 2007).

Colombia is the fourth largest palm oil producer in the world (Payán & Boron 2019; Boron *et al*. 2019), and the main producer in America with about 540,687 ha cultivated in 2018 [Federación Nacional de Cultivadores de Palma de Aceite (Fedepalma) 2019], but also is the country with the second richest bat fauna with 209 species (Ramírez-Chavez *et al.* 2020). The effect of these agricultural systems on bat diversity is understudied, especially in the regions where the extent of oil palm plantations have been growing exponentially in the country. The biogeographical region of the Orinoquía in Colombia is currently one of the main areas of oil palm expansion in the country, replacing to a greater extent pastures, rice plantations and natural savannas (Ocampo-Peñuela *et al.* 2018; Pardo *et al*. 2015; Castiblanco *et al.* 2013; Etter *et al*. 2011).

Many studies have focused on analysing differences in community structure and species composition between natural habitats (i.e., forests and savannas) and oil palm agricultural areas of several taxa such as birds (Bennett *et al.* 2018; López-Ricaurte *et al.* 2017), arthropods (Turner & Foster 2009), herpetofauna (Gallmetzer & Schulze 2015; Faruk *et al.* 2013) medium-and large-sized mammals (Pardo *et al.* 2018a; Pardo & Payán 2015), and bats (Freudmann *et al.* 2015; Nur-Juliani *et al.* 2011). Overall, in comparison with natural habitats, oil palm plantations show a striking reduction in species richness and shifts in species composition, removing rare and specialist species, and favouring generalists. Some variables like spatial heterogeneity, vegetation complexity, proximity to forest, and the history of coverage transformation can influence the assemblages in oil palm monocultures.

Since the Colombian government declared that palm oil plantations are one of the most important economic sectors for the country (Iragorri-Valencia 2016), oil palm agriculture probably will increase rapidly in coming years, despite the uncertainty about its effects on biodiversity and ecosystem services (Pardo *et al.* 2015). Characterising the structure and composition of species assemblages in oil palm plantations is therefore important in the contribution of basic information to better understand the possible impacts of these agricultural areas on biodiversity, which can be useful for future conservation planning. Here, we aimed to describe the structure and composition of bat assemblage in an oil palm plantation in the Colombian Llanos foothills by assessing how bat diversity differed between secondary forest and palm plantations. We discuss the implications of palm production for supporting bats and the possible effects of these plantations on the ecosystem services provided by these flying mammals.

## MATERIALS AND METHODS

### Study Area

The study was carried out in the Hacienda La Cabaña plantation (centroid of the study area: 4°18′18.4″ N, 73°21′26.5″ W), an oil palm agricultural area situated in Cumaral municipality (Meta Department), located in the Colombian Llanos foothills, between 310 m and 368 m elevation. The average annual rainfall ranges from 2500 mm to 3500 mm, the average annual temperature is 21°C and the relative humidity is 84%. The dry season corresponds to the months of December to March and the rainy season from April to November (Municipio de Cumaral 2009). The oil palm plantation has an area of 2,200 ha cultivated in African palm (*Elaeis guineensis*). Within the plantation, there are immersed forest remnants; and outside of it are livestock pastures, secondary vegetation and gallery forests (Cely-Gómez & Castillo-Figueroa 2019).

### Bat Sampling

**Figure 1:**
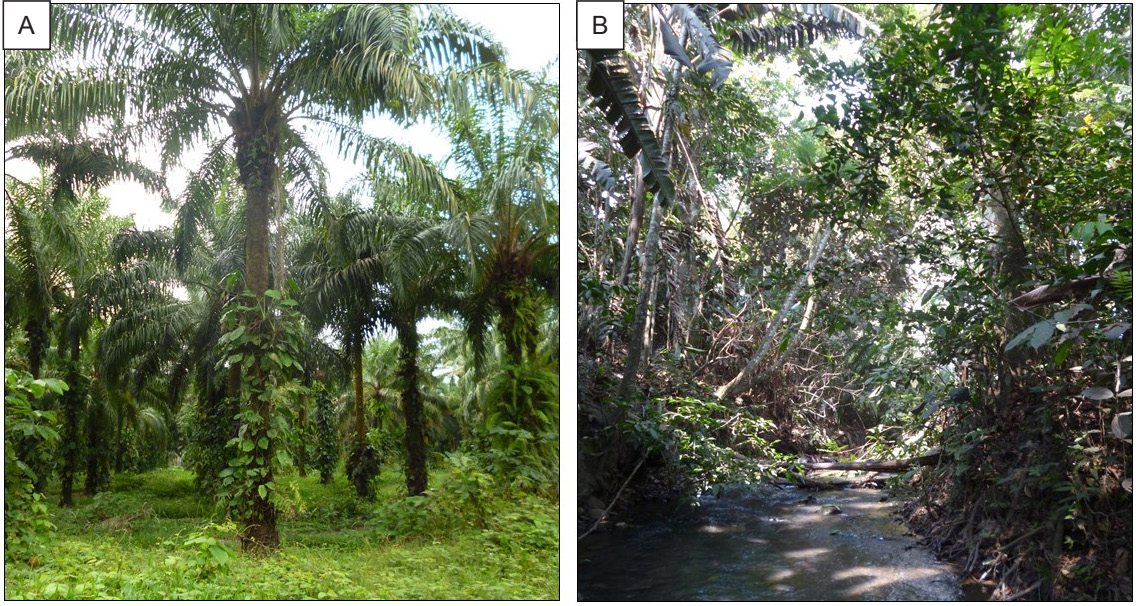
Two habitats were sampled within this agricultural area: (A) oil palm crops and (B) secondary forest.

Sampling was conducted during January and February 2016 at the end of the dry season. Due to the heterogeneity between the heights of palms and forest, a stratified sampling was made, where each palm plantation coverage corresponded to three different height (5 m, 15 m and 20 m) (Fig. 1A) and we compared it to a secondary forest area within the palm matrix (Fig. 1B). The distance between the sampling sites ranges from 1 km to 2 km.

We captured bats in each coverage with seven 6 m × 3 m mist-nets and one 9 m × 3 m mist-net, which were opened between 18:00 and 06:00. The sampling effort was calculated as the ratio of the product of the mist-net meters and the sampling hours on the sampling nights (m^2^/h mist-net) according to Estrada-Villegas *et al.* (2010). We sampled a total of 12 effective nights, three in each of the four coverages (forest, oil palm plantations heights 5 m, 15 m and 20 m). The effort for all sampling nights was 1,620 m^2^ mist-net hrs.

We recorded sex, reproductive status and standard morphometric measurements of captured bats (Kunz & Parsons 1988). We individually marked each bat by punch-marking numbers into their wing membranes with tattoo pliers for small domestic animals (Bonaccorso & Smythe 1972), prior to release. We collected one voucher specimen for each morphospecies captured to confirm the identifications of individuals captured. Collections were made under the permit from the National Authority of Environmental Licenses (Autoridad Nacional de Licencias Ambientales, ANLA) (Ministry of Environment and Sustainable Development, Resolution No. 0546; ANLA filing mobilisation permit: 2017011724-1-000). We deposited voucher specimens in the mammal collection of the *Museo Javeriano de Historia Natural* (MPUJ) (MPUJ-MAMM 2312-2334). Identifications of the individuals captured were made according to Gardner (2007), and for the genus *Platyrrhinus* the classification of Velazco (2005) was followed. Due to the difficulty separating *Carollia perspicillata* from *Carollia brevicauda* based on external characters measured in the field, these species were grouped as *Carollia* spp. (Cely-Gómez & Castillo-Figueroa 2019; Marciante *et al.* 2015).

### Data Analysis

We calculated the representativeness of bat species for the whole assemblage and for each coverage, by the first order Jackknife estimator. To eliminate the effect of the sequence in which each individual bat was added, we randomised sample order (*n* = 100) using EstimateS 9.1 (Colwell 2013). The structure of bat assemblage was described based on the effective number of species (Jost 2006), which corresponds to three diversity measures: (1) order 0 diversity (*q*^0^ = estimated species richness), (2) order 1 diversity (*q*^1^ = effective numbers of species taking into account proportional abundances in the assemblages), and (3) order 2 diversity (*q*^2^ = effective numbers of species according to the abundances of rare and dominant species); calculated with SPADE software (Chao & Hsieh 2015). Additionally, the Pielou evenness index (*J*′) was calculated using PAST 3.01 software (Hammer *et al.* 2001). The indices were compared between coverages (forest vs. oil palm) through bootstrapped 95% confidence intervals. Finally, we constructed rank-abundance curves in order to describe the bat assemblage structure at each coverage.

## RESULTS

**Figure 2:**
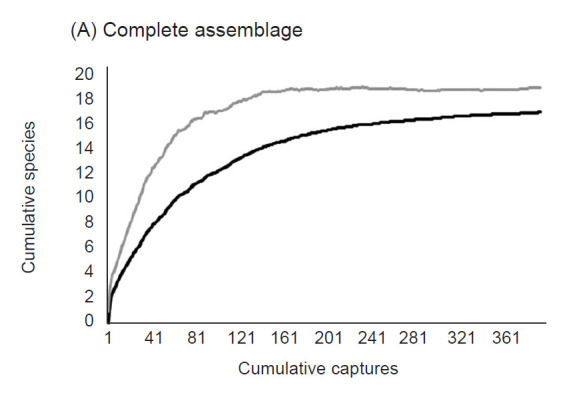

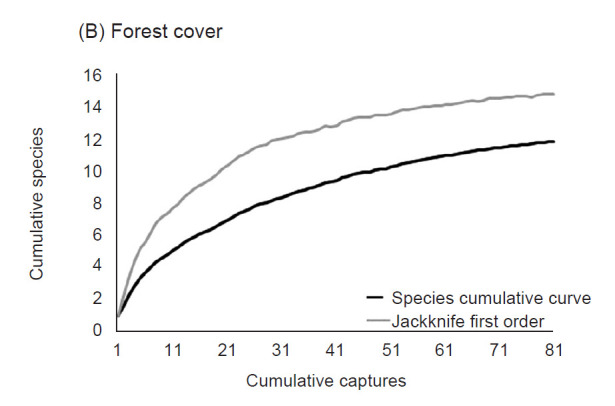

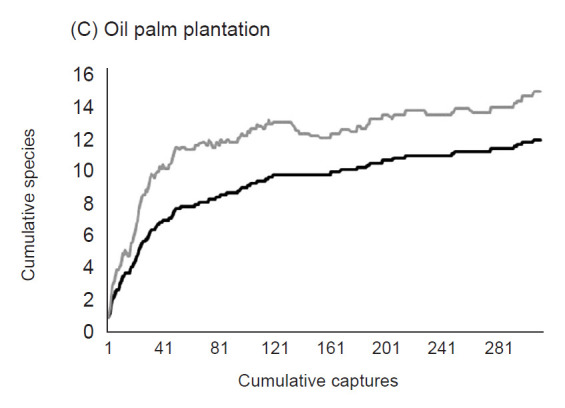
Accumulation curves for: (A) the whole assemblage, (B) for the forest coverage, and (C) for the oil palm plantation. Obtained at the Hacienda La Cabaña farm between January and February 2016. Y-axis represents cumulative species, X-axis the cumulative captures. The grey line shows the first order Jackknife and the black line the observed data for each analysis.

We captured 393 bats and recaptured 17 individuals for a total of 410 captures of 18 species and three families (Table 1). Phyllostomidae had the highest species richness (S = 16), and Vespertilionidae and Emballonuridae were each represented by a single species (*Myotis nigricans* and *Saccopteryx bilineata*, respectively). From Phyllostomidae, the Stenodermatinae subfamily presented eight species (44%); Carollinae and Phyllostominae had four species each (44%); and Desmodontinae and Glossophaginae a single species each (12%). Phyllostomidae was the most dominant family with 386 individuals (98.2%). *Carollia* spp. (*C. brevicauda* + *C. perspicillata*) represented 56.7% of the captures (*n* = 223), followed by *Artibeus planirostris* (*n* = 94, 29.9%). The assemblage as a whole presented a Pielou evenness index *J′* = 0.52 and the diversity values were: *q*^0^ = 18.0, *q*^1^ = 4.3 and *q*^2^ = 2.6. The species accumulation curve got to an asymptote (Fig. 2A) and with Jackknife estimator has the representativeness of 89.5%.

**Figure 3:**
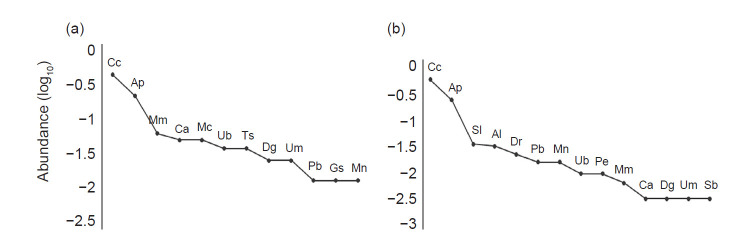
Abundance range curves for: (a) the forest and (b) the plantation. Species codes as follows: *Carollia* spp. (Cc), *Artibeus planirostris* (Ap), *Sturnira lilium* (Sl), *Artibeus lituratus* (Al), *Desmodus rotundus* (Dr), *Platyrrhinus brachycephalus* (Pb), *Myotis nigricans* (Mn), *Uroderma bilobatum* (Ub), *Phyllostomus elongatus* (Pe), *Mesophylla macconnelli* (Mm), *Carollia castanea* (Ca), *Artibeus* cf. *gnomus* (Dg), *Uroderma magnirostrum* (Um), *Saccopteryx bilineata* (Sb), *Mimon crenulatum* (Mc), *Tonatia saurophila* (Ts), *Glossophaga soricin*a (Gs).

Three species were found exclusively in secondary forest (*Glossophaga soricina, Mimon crenulatum* and *Tonatia saurophila*), while five were unique to oil palm plantation (*Desmodus rotundus, Saccopteryx bilineata, Phyllostomus elongatus, Sturnira lilium* and *Artibeus lituratus*) for a total of 13 and 15 species, respectively. We captured 81 individuals in the forest and 312 individuals in the oil palm plantation (Fig. 3). *Carollia* spp. and *Artibeus planirostris* were the dominant ones in both oil palm plantation and forest (Table 1). For each coverage the accumulation curve got to an asymptote (Figs. 2B and 2C) and with Jackknife estimator has the representativeness of 80.2% for forest and 80% for oil palm plantation. Diversity *q*^1^ and evenness (*J′*) were higher in forest than oil palm, but the other dimensions of diversity, including species richness (*q*^0^) and diversity (*q*^2^), showed no significant difference between coverages (Table 2).

## DISCUSSION

Our study presents a scenario of oil palm expansion in a Neotropical context where bats are prone to change their assemblage, facilitating the establishment of generalist species. Most of the research has been conducted mainly in the Paleotropics, where greater abundance of generalist species has been found in oil palm agricultural areas in comparison to forest areas (Syafiq *et al.* 2016; Nur-Juliani *et al.* 2011; Fukuda *et al.* 2009; Danielsen & Heegaard 1995). However, some investigations carried out in Costa Rica have found changes in the structure and composition of bat assemblages with an increase in the abundance of Stenodermatinae species in oil palm plantations when compared to forest cover (Freudmann *et al.* 2015). Our results show that generalist bats (*Artibeus* sp., *Carollia* spp.) increased their relative abundance in oil palm plantations (Fig. 3), as has been reported in different agricultural systems such as coffee (Kraker-Castañeda & Pérez-Consuegra 2011; Numa *et al.* 2005), rubber-cacao plantations (Heer *et al.* 2015) a mixed citrus plantations and banana plantations (Estrada & Coates-Estrada 2002). To our knowledge, this is the first study to describe bat assemblages in different coverages associated with oil palm plantations in Colombia.

Species representativeness of 89.5% indicates a high completeness of sampling for the bat assemblage in the study site (Fig. 2A). The species of *Carollia* spp., *S. lilium*, *A. lituratus* and *A. planirostris* were the most abundant in the oil palm plantation (Fig. 3), which can be explained because of the high flexibility in habitat use combined with functional traits such as body size (in the case of *Artibeus* sp.), high mobility and dietary flexibility (Cely-Gómez & Castillo-Figueroa 2019; Castillo-Figueroa & Pérez-Torres 2018; Freudmann *et al.* 2015; Kraker-Castañeda & Pérez-Consuegra 2011; Saldaña-Vásquez *et al.* 2010). We detected significant differences in the diversity (*q*^1^) and evenness (*J′*), which may be due to the abundances of the dominant species in each of the coverages as is reflected in the range-abundance curves (Fig. 3).

Even though the estimators did not show significant differences in species richness between the coverages, the breadth of the confidence interval for oil palm plantation is indicating a greater potential in the number of species for this coverage (Table 2), which contrasts with other studies from Southeastern Asia (Syafiq *et al.* 2016; Danielsen & Heegaard 1995). This result may be related to the coverage change, since in the study area there was a change from pastures to plantations, and this could increase the connectivity with other forest units of the landscape by using the plantations as stepping stones (Freudmann *et al.* 2015). Moreover, as the ages and heights of the plantations were not homogeneously, this can generate a greater variability of coverage in the total plantation that reduce the predation risk for bats compared to pasture areas (Estrada *et al.* 1993).

The presence of *Desmodus rotundus* exclusively in the plantation areas, can be explained by livestock and domestic animals within palm plantations, which increases the availability of food (Brown 1994). Other species such as *Saccopteryx bilineata* and *Phyllostomus elongatus* were also captured only in palm crops, possibly because the former is a common resident of human buildings (Yancey *et al.* 1998), many of which were near to the plantations in our study area, and the latter is an omnivore bat characterised by high feeding plasticity that can persist in human-modified landscapes (Morales-Marínez *et al.* 2018). On the other hand, *Glossophaga soricina* was recorded only in the forest coverage (Table 2). This nectarivorous species has shown high abundances in agricultural systems such as coffee plantations or mixed plantations that have a greater supply of food and shelter compared to oil palm plantations (Numa *et al.* 2005; Estrada & Coates-Estrada 2002; Castaño 2009). Likewise, *Gardnerycteris crenulatum* and *Tonatia saurophila* were captured exclusively in the forest, probably because both are gleaning bats adapted to foraging in habitats characterised by complex vegetation, with hollow trees and dense foliage for roosting sites (Cleary *et al*. 2016). As we mentioned before, in our study area oil palm plantations did not replace forest cover but pastures, as has been typically in the Colombian Eastern region (Etter *et al.* 2011). Pastures have low conservation value for several biological groups in Colombia (Payán & Boron 2019; Boron *et al*. 2019; Gilroy *et al*. 2015), therefore replacing pastures with oil palm plantations can reduce for some species the isolation distances between forest patches embedded in human-dominated matrix (Freudmann *et al.* 2015).

Other biological organisms such as arthropods, birds, amphibians and reptiles have been compared between oil palm plantations and pastures, thus finding similar or higher species richness in the former (Gilroy *et al.* 2015). For medium and large mammals, a greater species richness has been found in riparian forests compared to oil palm plantations (Pardo *et al.* 2018a; Pardo & Payán 2015), although for some generalist mesocarnivores (*Cerdocyon thous*), and anteaters (*Myrmecophaga tridactyla*) may persist in oil palm plantations with high abundances (Pardo *et al.* 2018b; Pardo *et al.* 2019). Despite the homogeneity of the oil palm plantations, they provide coverage, unlike pastures, which can be beneficial for some bat species by decreasing their risk of predation and by ease movement between habitat patches, such as flight corridors. Studies conducted in the Neotropics, mention that maintaining forest cover within the plantation favors the biodiversity of the system by generating a “spillover effect” towards the plantation areas (López-Ricaurte *et al.* 2017; Lucey *et al.* 2014; Freudmann *et al.* 2015; Gilroy *et al.* 2015), which in the case of some bats contributes to the reduction of species isolation and, probably the maintenance of ecosystem services provided by them. For example, dominant species of fruit bats (*A. lituratus*, *A. planirostris*, *S. lilium*, *Carollia* spp.) may play a key role in the ecological restoration in these agricultural areas, for which the conservation of forest areas is necessary in order to maintain these species (Cely-Gómez & Castillo-Figueroa 2019).

Due to the increase in oil palm plantations in the coming years in Colombia (Ministerio de Agricultura y Desarrollo Rural 2006), it is fundamental to improve the management practices of this productive system to avoid profound negative impacts on biodiversity (Pardo & Campbell 2019). Considering the few protected areas recognised by the government in the Orinoquía region of Colombia, the conservation mechanisms of the native forest should also be supported by landowners (Castillo-Figueroa *et al.* 2019; Pardo *et al.* 2018a: Pardo & Campbell 2019). For example, polyculture farming in oil palm smallholdings provides roosting sites for frugivorous bats because of the additional plantations (e.g., *Musa* spp., *Manihot esculenta, Zea mays, Mangifera indica*) and trees planted alongside the oil palm plantations (Syafiq *et al.* 2016). In other mammal groups, it has been suggested that allowing undergrowth vegetation within oil palm plantations and maintaining a greater habitat heterogeneity in the landscape, including also forest patches and riparian corridors can increase the probability of occurrence of medium and large mammals in oil palm landscapes (Pardo *et al.* 2019). The creation of buffer zones has been also proposed as a wildlife-friendly practice that can facilitate the movement of animals reluctant to use oil palm agricultural areas (Freudman *et al.* 2015). These types of approaches should be considered for palm production in a sustainable way that drives economic growth, as well as biodiversity conservation in the Colombian Orinoquía.

## CONCLUSION

In conclusion, even though forest coverage had a higher diversity in *q*^1^ and evenness (*J′*) than the oil palm plantation, there was no difference in species richness (*q*^0^) and diversity (*q*^2^) between the two habitat types. However, the species abundance between these habitat types was very different with *Artibeus* spp. and *Carollia* spp. as the most dominant ones in oil palm plantations. Considering the growth of oil palm production in Colombia, before making any forest management decisions is urgent to better understand the ecological impacts of this productive system in biodiversity in order to improve the management practices and avoid irreversible effects on Colombian Llanos ecosystems.

## Figures and Tables

**Table 1:** Bats captured at Hacienda La Cabaña plantation (Meta, Colombia) between January and February 2016.

Family	Subfamily	Species	Abundance		

			Secondary forest	Oil palm plantation	Both habitats
Emballonuridae		*Saccopteryx bilineata*		1 (0.3)	1 (0.3)
Phyllostomidae	Phyllostominae	*Gardnerycteris crenulatum*	4 (4.9)		4 (1)
		*Phyllostomus elongatus*		3 (1)	3 (0.8)
		*Tonatia saurophila*	3 (3.7)		4 (0.8)
	Stenodermatinae	*Artibeus lituratus*		10 (3.2)	10 (2.5)
		*Artibeus planirostris*	18 (22.2)	76 (24.4)	94 (23.9)
		*Artibeus* cf*. gnomus*	2 (2.5)	1 (0.3)	3 (0.8)
		*Mesophylla macconnelli*	5 (6.2)	2 (0,6)	7 (1.8)
		*Platyrrhinus brachycephalus*	1 (1.2)	5 (1.6)	6 (1.5)
		*Sturnira lilium*		11 (3.5)	11 (2.8)
		*Uroderma bilobatum*	3 (3.7)	3 (1)	6 (1.5)
		*Uroderma magnirostrum*	2 (2.5)	1 (0.3)	3 (0.8)
	Carollinae	*Carollia castanea*	4 (4.9)	1 (0.3)	5 (1.3)
		*Carollia* spp.*	37 (45.7)	186 (59.6)	223 (56.7)
	Desmodontinae	*Desmodus rotundus*		7 (2.2)	7 (1.8)
	Glossophaginae	*Glossophaga soricina*	1 (0.2)		1 (0.3)
Vespertilionidae		*Myotis nigricans*	1 (1.2)	5 (1.6)	6 (1.5)

	Total		81 (20.6)	312 (79.3)	393 (100)

*Note*: In brackets the percentage of relative abundance in the habitat. Carollia spp. = + C. perspicillata + C. brevicauda.

**Table 2:** Effective numbers of species and evenness for bats from Hacienda La Cabaña plantation (Meta, Colombia).

Index	Secondary forest				Oil palm plantation		

	LCI	Mean value index	SCI		LCI	Mean value index	SCI
Diversity *q*^0^	12.7	14.7	22.2	=	14	17.2	36.9
Diversity *q*^1^	4.6	5.7	6.9	>	3.1	3.6	4.1
Diversity *q*^2^	2.5	3.6	4.7	=	2.1	2.3	2.6
Pielou (*J′*)	0.62	0.7	0.79	>	0.45	0.40	0.54

*Note*: LCI = Lower confidence interval, SCI = Superior confidence interval.
